# Cross-Cultural Notions of Risk and Liberty: A Comparison of Involuntary Psychiatric Hospitalization and Outpatient Treatment in New York, United States and Zurich, Switzerland

**DOI:** 10.3389/fpsyt.2018.00267

**Published:** 2018-06-19

**Authors:** Florian Hotzy, Jeff Kerner, Anke Maatz, Matthias Jaeger, Andres R. Schneeberger

**Affiliations:** ^1^Department for Psychiatry, Psychotherapy and Psychosomatics, University Hospital of Psychiatry Zurich, Zurich, Switzerland; ^2^Montefiore Medical Center, Bronx, NY, United States; ^3^Department of Psychiatry and Behavioral Sciences, Albert Einstein College of Medicine, New York, NY, United States; ^4^Psychiatrische Dienste Graubünden, Chur, Switzerland; ^5^Universitäre Psychiatrische Kliniken Basel, Universität Basel, Basel, Switzerland

**Keywords:** coercion, involuntary hospitalization, assertive outpatient treatment, legislation, clinical culture, severe mental illness

## Abstract

Involuntary hospitalization is a frequently discussed intervention physicians must sometimes execute. Because this intervention has serious implications for the citizens' civil liberties it is regulated by law. Every country's health system approaches this issue differently with regard to the relevant laws and the logistical processes by which involuntary hospitalization generally is enacted. This paper aims at analyzing the regulation and process of involuntary hospitalization in New York (United States) and Zurich (Switzerland). Comparing the respective historical, political, and economic backgrounds shows how notions of risk and liberty are culture-bound and consequently shape legislation and local practices. It is highly relevant to reconsider which criteria are required for involuntary hospitalization as this might shape the view of society on psychiatric patients and psychiatry itself. Furthermore, this article discusses the impact that training and experience of the person authorized to conduct and maintain an involuntary hospitalization has on the outcome.

## Introduction

The use of coercion—whilst deeply controversial and ethically problematic—is a global phenomenon in psychiatry. It comes in many different forms, the most common ones being involuntary hospitalization, forced administration of medication, confinement in seclusion, physical or mechanical restraint, and compulsory treatment in outpatient settings. As all coercive measures severely compromise a person's autonomy and right to freedom, most nations have strict codes regulating their application (1–4)^1^. Almost all over the developed world, the standard for involuntary hospitalization, as well as for installing involuntary outpatient treatment, is that the person must have a mental illness and/or represent an acute threat to him- or herself or to others, and that less coercive measures (e.g. acute outpatient crisis intervention, voluntary admission, home treatment) have failed (1, 3). The specifics of legal regulation, clinical practice and societal attitude toward coercion, however, vary greatly between different regions of the globe (1, 3, 5). It is well known that internationally, the rate of involuntary hospitalizations fluctuates enormously (6, 7). Interestingly, large differences in the rate of involuntary hospitalizations can not only be found between culturally very different regions, but also between countries with comparable legislation and attitudes toward coercion (1): for example, Denmark and Portugal have the lowest rates of involuntary hospitalizations in Europe, although the relevant legislation in the two countries differs considerably. In contrast, the legislation and cultural characteristics of Denmark and Finland are less dissimilar, but the latter appears to have one of the highest rates of involuntary hospitalizations in Europe (1). Important differences can even be found within a single country, as is the case in Switzerland (8). It is thus not entirely clear to what extend legislation and cultural aspects account for specific differences in the implementation of coercive measures (2, 6).

Sharing concerns about the recent rise in the use of coercion in psychiatry worldwide (9), and hoping ultimately to contribute to its reduction, we think it is important to gain a deeper understanding of the legal and socio-cultural factors underlying the use of coercive measures. Based on our own experience as practicing psychiatrists in two different regions of the globe, namely New York, U.S., and Zurich, Switzerland, this paper offers a comparative analysis of the laws regulating coercion and of the actual clinical practices in these two regions.

After a short account of the respective histories of coercion and of the current organization of mental health care provision in the U.S. and Switzerland, we then compare the legal regulations surrounding coercion: specifically, involuntary hospitalization and involuntary outpatient treatment in New York and Zurich. As far as the available data allow, we also compare the actual clinical practice of involuntary hospitalization and involuntary outpatient treatment. We end with a discussion of the differences and similarities found, trying to better understand how the above-mentioned factors, especially notions of risk and liberty, are interrelated and converge in shaping the ‘culture of coercion’ and related research in the two regions.

## Cultural and historical backgrounds

Both the United States of America (U.S.) and Switzerland significantly value civil liberties. This sentiment is also reflected in the respective constitutions. The preamble to the U.S. Constitution from 1787, specifies that its aim is to “secure the Blessings of Liberty to ourselves and our Posterity” (10). The analogous section in the Swiss Constitution, revised in 1999, states “The Swiss People and the Cantons, mindful of their responsibility toward creation, resolved to renew their alliance so as to strengthen liberty, democracy, independence and peace…” (11). Against this cultural and legal background, the notion that citizens could be held against their will without having been formally accused with reasonable evidence of a crime is of great concern. Nevertheless, both countries' psychiatric establishments have checkered histories in this regard and have been associated with widespread public abuse.

In the U.S., psychiatrists in the early Twentieth century administered several interventions that were harmful to psychiatric patients, and were largely carried out without permission and informed consent. Examples of such practices were pyrotherapy (a practice by which infectious agents were transfused to psychiatric patients with the aim of inducing a fever that was thought to cure the mental illness), insulin shock therapy (inducing hypoglycemia, often resulting in coma), and the prefrontal lobotomy (12). Electroconvulsive therapy, which is still thought to be an effective treatment for several psychiatric disorders (13, 14) and goes on being used despite its side effects (esp. memory loss) (15), was often used without the proper sedatives and anesthetics, and sometimes used as a punitive measure. These interventions were dangerous, ineffectual, and consequently received large amounts of public criticism and led to general mistrust of the institution of psychiatry.

Switzerland, in turn, has its own history of abusive psychiatric practices. Eugenics was discussed in psychiatric circles and sterilization and castration were performed in psychiatric hospitals (16). In 1928, the Grand Council of the Canton Vaud amended the 1901 law called, “Treatment of Persons Affected by Mental Illnesses.” The law was changed to include the idea that “A person affected by a mental illness or injury can receive medical intervention to prevent reproduction if the person is considered incurable and if it is likely that the progeny will be defective.” (16). With the family tree of the so called “family zero” and concomitant theories about eugenics the first clinic director of the psychiatric clinic in Graubünden (one canton of Switzerland) was often used as a reference for the atrocities during Nazism (17).

Eugenics lost favor among Swiss psychiatrists and the population in general in the late 1930's and early 1940's, when reports of forced sterilization in Germany reached other parts of Europe (16). Although this led to changes in the attitudes, the venerability of the psychiatric institution in Switzerland was already compromised. Further damaging psychiatrists' reputation in Switzerland was caused by the poor condition of asylums in the early Twentieth century, which were described to be more or less prison-like. During these years, patients were admitted to psychiatric hospitals against their will at a rate of 97% (16).

Overall, the use of coercive measures in psychiatry results from the negotiation of three values: safety of the community, need for treatment, and protection of the patient's liberties (18). Following from the history of abuse in psychiatry and the influence of the anti-psychiatric movement in the 1960‘s (19), there was a move toward patients’ rights, i.e. the emphasis of every patient's right to freedom and to execute their own will. This can be seen in changes of the law and of organizational structures in different countries and these changes have indeed led to a reduction of coercive treatment (18, 20). However, the emphasis on respecting patients' “rights, will and preferences” (21) raised questions whether it is ethically correct to “withhold” treatment to patients, which was described as “dying with their rights on“(22). In the course of deinstitutionalization and changes in the law to encourage the autonomy of patients, length of stay has been significantly reduced, but on the other hand rates of (re)-hospitalizations (including involuntary hospitalization) increased (7, 23, 24), leading to the so called “revolving-door-psychiatry” (25). Critics of these trends found doubtful support by murder perpetrated by individuals who had received inadequate psychiatric care for their respective disorders. Some of these tragedies led to renewed media attention and public discussion emphasizing the public's need for protection (26). Thus, in some countries public opinion may have swung in the favor of increased safety in the community (27–29). This change in attitude led in turn to more authority for physicians to hold patients against their will if they are perceived to be a danger to themselves or others (29). The public discussion and especially media-coverage of such tragic events have an impact on the perception and stigmatization of psychiatric disorders in society (28).

## The economic backgrounds and healthcare regulations

While sharing some key features, Switzerland and the U.S. are significantly different countries with some profound distinctions in regards to the health care delivery systems (30, 31) and other features. Switzerland is a land-locked country of approximately eight million people, compared with the U.S. population of over 315 million citizens. However, both are to a certain extent de-centralized, with the 50 states in the U.S. and 26 cantons (similar to states in the U.S.) in Switzerland reserving legislative authority not specifically stipulated by the federal government. Both countries are affluent, and commit a relatively large proportion of their Gross Domestic Product (GDP) to health care. In 2014, Switzerland spent 11.7% of their GDP on health care, compared to the US' 17.1% in the same year (32).

In the U.S., the 2010 Affordable Care Act (ACA) changed the health care laws. This enacted a system whereby the majority of US citizens is required to purchase private insurance, whereas the indigent population is covered by the joint federal/state program Medicaid, and the elderly (over 65) are covered by Medicare. Since enactment of the law in 2010 the uninsured rate has declined from 16.0 to 9.1% in 2015 (33). In 2017 the newly elected president of the U.S. declared the goal to revise the ACA and even abolish it in favor of a new health care system called the Better Care Reconciliation Act (BCRA) leading to a potential reduction of costs but also to an increase of uninsured persons (34, 35).

In Switzerland, as stipulated by the 1996 Health Insurance Law, all residents are required to purchase private insurance. Government assistance is available in cases where residents are unable to afford an insurance package so that every resident has health insurance (36).

On the organizational level the number of beds in mental health hospitals decreased dramatically in the US during the last decades to a mean of 12 beds per 100.000 inhabitants (min 3.5 in Minnesota, max 42 in the District of Columbia) (37), compared to 72 in Switzerland (38). Health policy experts call attention to low number of beds in the US, as a minimum of 50 beds per 100,000 people is considered necessary to provide minimally adequate treatment for patients with mental illness (37). Also the number of psychiatrists in private practice differs considerably between the countries, with Switzerland having one of the highest number of psychiatrists per 100.000 citizens (39). Specifically, compared to the US, Switzerland has four times the amount of psychiatrists in private practice. Focusing on New York State and the Canton of Zurich, the differences are even more pronounced, with Zurich having 6 times more psychiatrists than New York State. For further details see Table 1.

**Table 1:** Mental health care supply in the US and Switzerland.

	**United States of America/New York**	**Switzerland/Zurich**	**References**
Psychiatrists per 100.000	12/11	41/60	(39–41)
Psychiatric beds in mental hospitals per 100.000	12-23/16	72/78	(37, 38, 42)
Outpatient facilities per 100.000	2 /^*^	5 /^**^	(43)
Admissions per 100.000	480/676	690/939	(42, 44–46)
Involuntary hospitalizations in Percent	26-51/^*^	4-37/25	(8, 47–49)

*Differences in the healthcare organization and rates of involuntary hospitalizations in the US and Switzerland and the states New York and Zurich are shown*.

* *No data available for the New York State*;

** *No data available for the Canton of Zurich*.

Both countries are a collection of states (cantons) with respective governing bodies. Each state's government is responsible for drafting laws not specified by the federal government. Neither Bern (the Capital of Switzerland), nor Washington, D.C. have specifically dictated the exact processes by which psychiatric patients are admitted involuntary to institutions, so each state and canton has distinct regulations.

It would go beyond the scope of this paper to compare the laws of each country. The aim was to examine two representative region's respective laws concerning compulsory hospitalization.

New York State and the canton of Zurich are especially populous regions in their countries, the United States of America and Switzerland. Both regions are anchored by the biggest cities in the two countries, namely New York City and Zurich, which are important cultural and economic centers in both countries and often considered the capitals of the respective countries, and neither is the seat of the federal government.

## Legislative regulation of involuntary hospitalization:

In New York State, the Office of Mental Health (OMH), under the ultimate jurisdiction of the Governor of New York State, dictates the laws regarding involuntary observation and hospitalization. These laws are called the Mental Hygiene Laws (MHL) and subsumed in Article 9 of the New York State Laws. There are essentially four laws (3) that govern this process based on the time period the patient is held and treated against their will: MHL 9.40 regards acute psychiatric emergencies. It states the patients may be treated in a Comprehensive Psychiatric Emergency Program (CPEP) if the patient is suspected to have a mental illness and “endangers him/herself or others (3).” After arriving at a CPEP the MHL 9.40 dictates that a staff physician (not necessarily a psychiatrist) must evaluate the patient within 6 h to determine if he or she indeed meets the standard delineated above. If so, a psychiatrist, must re-evaluate the patient within 24 h of the patient's presentation to confirm the original physician's assessment. If the second physician confirms the first physician's assessment, the patient must be transferred to an extended observation bed. The patient may remain there for up to 72 h, and then will either be discharged or converted to longer-term involuntary status covered by MHL 9.27 or 9.39. MHL 9.39 can follow after MHL 9.40 or be initiated independently (3). Again, a staff physician of the hospital (not necessarily a psychiatrist) must evaluate the patient and confirm that he or she has a mental illness and may be a harm to him/herself or others. Within 48 h, a staff psychiatrist must confirm the first physician's assessment. Similarly to MHL 9.40, if no such assessment is made, the patient must be discharged. If the psychiatrist does confirm the original physician's assessment, the patient is held on an inpatient psychiatric unit for up to 15 days. If two licensed physicians state that a longer involuntary hospitalization than 15 days seems to be necessary the MHL 9.39 may be converted to 9.27 status. To apply MHL 9.27 the patient must have a mental illness and pose a substantial threat of harm to self or others (3). The 9.27 status is valid for 60 days. After 60 days, if the director of a mental health facility believes the patient to be in need of further involuntary hospitalization, the process is referred to as “continued retention” and governed by the law 9.33 (3). This process requires a court hearing, whereby the clinic director submits the patient record to a mental health court and argues the need for continued involuntary treatment. At any point in the process of involuntary observation and hospitalization, the patient has the right to appear before a New York State Judge, with representation by a lawyer.

The corresponding laws in Switzerland have experienced recent changes. On January 1st, 2013 a new legislation on child and adult protection was implemented, stipulating the process of “care-centered placement” (“Fuersorgerische Unterbringung”) which is similar to the concept of involuntary hospitalization (2). Based on Article 426 of the Swiss Civil Code, a person who suffers from a mental disorder, mental disability or severe neglect may be placed in an appropriate facility if necessary treatment or care cannot be provided otherwise. In addition to hospitals and psychiatric clinics, nursing homes, and group homes can be chosen as a facility for “care-centered placement”. Involuntary hospitalization is only permitted if there is no less intrusive method available and an appropriate facility is accessible. The lack or presence of a patient's decision making capacity is, however, not a relevant criterion to determine an involuntary hospitalization. And also the criteria of danger to self or others are not required by law. Thus, theoretically a patient can be involuntary hospitalized without meeting the danger criterion. Article 426 also states that it must be taken into consideration whether the affected person is a burden to his or her environment (50). The canton Zurich has determined that besides the child and adult protective services (Kindes- und Erwachsenenschutzbehoerde, KESB), physicians who are licensed to practice medicine in Switzerland are authorized to determine the need for involuntary hospitalization. Physicians working for the institution to which the patient is admitted are not eligible to determine an involuntary hospitalization. Article 430 of the Swiss Civil Code determines that the physician issuing the certificate for involuntary hospitalization has to examine the patient personally and cannot base his decision on third party information. People who enter psychiatric inpatient treatment on voluntary bases and wish to leave the clinic can be retained by the clinical director or his deputy for a maximum of 3 days if they are a threat to themselves or others (thus, the danger criterion is required in this situation). After expiration of this deadline, the patient must be discharged unless a new certificate regarding an involuntary hospitalization according to article 426 of the Swiss Civil Code has been issued. The physician issuing this involuntary hospitalization must be board certified in psychiatry and not affiliated with the clinic. A certificate for involuntary hospitalization issued by a physician is limited to a maximum of 6 weeks unless the KESB has issued an extension, which then must be reviewed at least every 6 months. The patient must be informed orally and in written form about the involuntary hospitalization and the right to appeal at the regional court^1^.

In the light of the differences regarding legislation between both regions, the question arises if it results in differences in the organizational level of mental health care and/or in different rates of involuntary hospitalization.

In Switzerland, the rates of involuntary hospitalization differ widely, ranging from 8% in the city of Basel (the second largest city in Switzerland) to 25–33% in Zurich (the canton with the biggest city in Switzerland) (8, 47, 48). For the New York State there is a lack of comprehensive data concerning the rate of involuntary hospitalization due to different court jurisdictions for state and local hospitals and private hospitals. However, a study from 1989 found that 26–51% of inpatient admissions in the U.S. of America were involuntary (49). For further details see Table 1.

## Legislative regulation of involuntary outpatient treatment

In New York, the law regarding outpatient commitment was revised in 1999 after the public became aware of dramatic incidents caused by people with untreated and decompensated mental illness. Concrete changes to the NYS Mental Health Law were implemented after a young woman (Kendra Webdale) was pushed in front of a subway and died. Due to this tragedy the law was named after this woman and is known today as Kendra‘s Law, characterized in Section 9.60 of the NYS Mental Health Law^2^. This law grants judges the authority to issue orders that require people who meet certain criteria to undergo psychiatric treatment regularly. Lack of adherence can lead to an involuntary removal from the community for an evaluation in an emergency department, which can take up to 72 h and may be followed by a possible involuntary hospitalization if patients meet certain criteria. Kendra's Law cannot require patients to take medication against their will. The law is intended to prevent violent episodes and allows family members or friends to petition the court to order assisted outpatient treatment (AOT), a coercive outpatient treatment, before a dangerous situation might occur. To qualify for AOT patients must be 18 years or older, have a mental illness, be unlikely to survive safely without supervision, have a history of non-adherence with treatment that has led to either hospitalization or incarceration twice in last 36 months, or resulted in dangerous behavior within 48 months (51). Also, potential patients must be unlikely to voluntarily participate in treatment, and likely to benefit from AOT. The idea of AOT is that patients are more likely to comply with treatment to avoid randomly being brought to the emergency department.

In Switzerland, Art. 437 of the new child and adult protective law stipulates that the cantons are responsible for regulating the implementation of involuntary outpatient treatment (52). In Zurich, a court order issued by the KESB enables mental health caregivers to compel the patient to take medication as prescribed and participate in regular psychiatric consultations. If the patient refuses to comply with treatment, the KESB or the therapeutic team is allowed to assertively outreach to the patient. However, involuntary hospitalization is only possible if the legal criteria described above are met and forced medication is not allowed in the outpatient setting.

## Discussion

The U.S. and Switzerland are both high income countries that invest significant resources in their mental health systems. Whilst their approaches to mental health care share many similarities, there are also important cultural differences. These characteristics concern the relevant legislation, organizational structures of mental health care, the health insurance system and expert views. Also, views of the public and socioeconomic aspects may have a substantial effect on these divergences. Those different aspects in turn influence the legislation and will be discussed in the following section.

### Involuntary hospitalization in practice and research

The legislation, which stipulates involuntary hospitalization in the two regions, share many similarities. The essence of what constitutes grounds for involuntary hospitalization remains a mental disorder. Besides this, there are several interesting distinctions. In the State of New York, the danger to self and others is a major criterion for involuntary hospitalization and its prolongation. In Zurich, this danger criterion is not required and only listed as an additional criterion after the primary criteria, i.e., weakness and need for protection. In general, the Swiss Laws have a broader range in the criteria for conduction of an involuntary hospitalization, especially as there is a specific provision directed at the issue of whether the patient would be a burden on the family or other caretakers in the community when deciding whether involuntary hospitalization in necessary. With these broader formulated criteria, Swiss laws have a more farsighted view of the challenges facing the treatment of mental illness, accepting that the risk of meeting the criteria for initiation of an involuntary hospitalization is higher in many patients. One argument in favor of this criterion is that if care is burdensome to others, there will likely be a worse prognosis for the patient and his/her family. Nevertheless the question arises how a physician can define when the point is reached that the burden for others justifies the restriction of an individual's freedom (in this case the patient). A Norwegian study showed that many referring physicians felt pressured by the patient's environment to order an involuntary hospitalization (53). In Zurich, every physician, independent of the specialization, is allowed to refer patients involuntarily. It can be assumed that experience with psychiatric emergency situations differs across various medical subspecialties. Physicians with less experience in such situations might arguably be more easily pressured or fear legal consequences, leading to faster involuntary hospitalizations. More broadly formulated criteria might further this tendency. Zurich Mental Health Laws state clearly that the referring physician must not be affiliated with the clinic where the patient is referred to. In contrast, in New York it is possible for the physician who makes that determination to work at the same institution where the patient is hospitalized, which might lead to a conflict of interest. First, admitting patients can represent a financial incentive for the institution, which then pays the physician's salary. A physician who generates more revenue for the hospital could be seen as a better employee, and could be rewarded either financially, or with more institutional esteem. In New York, two physicians must agree that a patient requires involuntary hospitalization, and they often are co-workers. Because conflict in the workplace is unpleasant, there may be an incentive to agree, instead of evaluating a patient without any bias whatsoever. On the other hand, the regulations in Zurich compared to New York's system can prove to be time-consuming and costly for the hospital, which has to recruit outside physicians to evaluate their own patients in order to retain them. Even if the hospital itself is not responsible for these costs, it is still taxing on the overall mental health care system.

In New York, the law limits the length of an involuntary hospitalization at several time points, and a prolongation must be actively appealed for by the physicians in charge or additional physicians. This is contrasted by the Zurich legislation where an involuntary hospitalization can remain for as long as 42 days. This regulation spares resources on one hand, whereas on the other hand the consequent lack of regular revisions at court may increase the risk for longer involuntary hospitalizations The only other limitation provided for is if the patient him/herself appeals for an earlier discharge in court. This may account for the longer duration of involuntary hospitalizations in Zurich (20 days) (54) relative to the average length of stay in New York (11 days) (46).

The low number of psychiatric beds in New York may actually be a reflection of the low median length of stay among psychiatric inpatients. A reduction of psychiatric beds has also been discussed in connection with an increase of psychiatric patients in the prison system (55, 56). However, these findings are inconclusive as other studies have shown contradictory results (57, 58) and emphasized the importance of considering other factors, such as globalization, migration, changes in the concept of traditional families, economic pressures, and changes in mental healthcare to play a role in the increasing number of homelessness and imprisonment among mental health patients (57).

### Assertive outpatient treatment in practice and research

The fear of untreated psychiatric patients and their potential actions seems to be higher in New York or the U.S. in general compared to Zurich or Switzerland. The easy accessibility of weapons in the U.S. has been discussed as a contributing factor to this fear (59). It may also be partly explained by dramatic and public cases of murder caused by people with psychiatric disorders. One noticeable result of these tragedies was the implementation of Kendra's Law^2^ and the spreading of AOT in New York. Nevertheless, the enactment of Kendra‘s Law was also influenced by the trend for deinstitutionalization, which had its origin in the 1960‘s, and resulted in a low number of psychiatric beds in New York.

Because of the controversy of AOT, research about its effects and possible adverse effects has become an important field in epidemiological research. One of the major criticisms is the lack of uniformity in the law (4). Each state has a substantial legal autonomy concerning how to implement the policies and allocate funds, so AOT patients may have vastly different experiences in different parts of the country. However, research found that symptoms of patients decreased in patients receiving AOT due to greater use of mental health care (60). This may be one reason for fewer completed suicides, fewer violent episodes, better socialization (61), fewer hospitalizations (62), and a reduction of the revolving-door phenomenon (63) in patients receiving AOT. On the other hand one review of three randomly controlled trials found no significant effect for AOT except for the reduced risk for victimization in the patients which were in the AOT group (64). Some arguments for the use of AOT might be that it results in less coercive treatment for the participant because the affected person is less likely to be involuntarily hospitalized or arrested (65). Patients themselves termed their experiences as “being on hold” and as a result not taking responsibility for their lives. Many patients described their quality of life as being diminished. However, similar accounts are described for involuntary hospitalization. Some patients on the other hand described feelings of safety and the benefit of fast accessibility to service (66, 67), and there were patients who preferred AOT compared to hospitalization (67). In summary, AOT may be one form of coercion to reduce the use of other forms of coercion. The positive effect on the patients‘ outcome can be seen to be caused by a better accessibility for mental health care treatment and not by coercion itself. Although positive effects could be shown, perspectives of the different stakeholders (patients, mental health workers) are varied. The long-term effect on the therapeutic relationship and influencing factors in the outcome of AOT would be interesting future research topics.

In Zurich, the legislation is more restrictive according to involuntary outpatient treatment and as a result involuntary outpatient treatment does not play a role in mental health care. Therefore, research aimed at involuntary treatment only focuses on involuntary hospitalization in institutional settings.

## Conclusion

New York and Zurich are both high-income regions in the Western world. They share basic democratic and liberal values, respect for human rights, personal freedom, and autonomy. This broad cultural similarity entails similar approaches to mental health care, and yet as we have shown, there are important differences with regards to the specifics of clinical practice, esp. involuntary hospitalization and assertive outpatient treatment. These differences become understandable when local culture, with its specific historical, economic and social background, is taken into consideration. Psychiatry's characteristic dual role—its duty to provide care for mentally ill persons on the one hand and the role as society's “safe-keeper” on the other—implies that there is a constant tension among the treatment needs of the individual, the safety needs of society, and the individual's liberties (18). This negotiation is open to (micro) cultural influences and shapes legislation and practice, which turns into a constitutive factor of local culture.

The focus on the danger criterion in New York might aggravate the stigmatization of psychiatric patients and the field of psychiatry itself. This is relevant as it was shown that besides self-stigmatization of patients also the stigma of psychiatry itself can be problematic. Some patients avoid psychiatric help because they do not want their family, friends, or employer to find out about their mental health problem (68). Changes in the legislation reducing the importance of the danger criterion compared to the relevance of treating psychiatric symptoms might be beneficial. First, focusing on psychiatric problems might be helpful in the process of de-stigmatization of psychiatric patients. Second, it might help to shift the perception of psychiatry in its characteristic dual-role (69) more to an institution for patients who are in need of and want to receive psychiatric treatment.

In Zurich, the less specific criteria for involuntary hospitalization might lessen such stigmatization. However, they might lead to higher rates of involuntary hospitalization. With a broader margin of legal interpretation, the influence of the health care provider increases. In Zurich, the quality of the commitment process and the course of treatment after involuntary hospitalization differs according to the referring physicians and their experience dealing with psychiatric emergencies (54, 70). Thus, restricting which physicians are authorized to hospitalize patients against their will (71), or better educating referring physicians, might be a promising model to reduce unnecessary involuntary hospitalizations. Also, the possibility of consulting with an experienced colleague might be helpful. Furthermore, regular, mandatory evaluations, and ongoing justification for the necessity of an involuntary hospitalization might prove beneficial. An independent civil court should have the authority to implement and supervise such regulations. To ensure that involuntarily hospitalized patients can protect their civil liberties, the law should require access to a free-of-charge legal counselor (7).

Given the gravity of coercion, knowledge of the laws that govern the process of coercive treatment locally and awareness of local alternatives to this most intrusive intervention appear to be crucial. Also, research examining ways to minimize the use of coercive interventions is fundamental. However, such research needs to be implemented in a specific cultural context. While certain “best practices” might be highly beneficial for one community, they might not be suitable for another. It is therefore essential to develop a sensitivity to cultural and local influences on psychiatric practice. A cross-cultural comparison can help develop such sensibility. It can also open one's eyes to alternative ways of meeting common challenges and thereby offer inspiration for change.

## Ethical statement

This study has been performed in accordance with the ethical standards laid down in the 1964 Declaration of Helsinki and its later amendments.

## Author contributions

FH, JK, AM, and AS: Conception and design; data collection, analysis, and interpretation; FH, JK, AM, MJ and AS: Drafting the article and revising it critically for important intellectual content.

### Conflict of interest statement

The authors declare that the research was conducted in the absence of any commercial or financial relationships that could be construed as a potential conflict of interest. The reviewer CM declared a past co-authorship with one of the authors MJ to the handling Editor.

## Footnotes

^1^Schweizerisches Zivilgesetzbuch (Erwachsenenschutz, Personenrecht und Kindesrecht) (2008).

^2^New York Consolidated Laws, Mental Hygiene Law. MHY § 9.60, Assisted outpatient treatment (2017) Available online at: http://codes.findlaw.com/ny/mental-hygiene-law/mhy-sect-9-60.html
